# Investigation of Dual Network Construction for Toughening in Bio-Based Polyamide Composites

**DOI:** 10.3390/polym16162248

**Published:** 2024-08-08

**Authors:** Chenxu Zhou, Chao Ding, Huaguang Yang, Xianbo Huang

**Affiliations:** 1 National Engineering Laboratory for Plastic Modification and Processing, Kingfa Scientific and Technological Co., Ltd., Guangzhou 510275, China; zhouchenxu@kingfa.com.cn (C.Z.); dingchao1@kingfa.com.cn (C.D.); yanghuaguang@kingfa.com.cn (H.Y.); 2 State Key Laboratory of Polymer Materials Engineering of China, College of Polymer Science and Engineering, Sichuan University, Chengdu 610065, China; 3 Institute of Emergent Elastomers, School of Materials Science and Engineering, South China University of Technology, Guangzhou 510641, China

**Keywords:** bio-based polyamide, dual network, compatibility, fluidity, toughness

## Abstract

This study investigated the role of constructing a dual network in toughening bio-based long-chain polyamide 610 (PA610) composites. Rheological studies were conducted to reveal the effects of toughening agent type and content on the material properties. According to the variation trend of mechanical properties and the appearance of a rheological low-frequency plateau of the materials, the percolation network concentration *ϕ*_c_ of the toughening agent in the PA610 matrix was determined to be 13.5 vol.%. The interfacial interaction of the composite was evaluated through the percolation theory, and the scaling value *v* = 1.36 for both indicated the good affinity between PA610 and the toughening agent. Rheology results found that the combination of ethylene terpolymer (PTW) and maleic anhydride-g-styrene-b-(ethylene-butylene)-b-styrene (MAH-SEBS) could achieve an optimal balance between the mechanical properties and fluidity of the composites. Furthermore, the addition of ultra-high-molecular-weight polytetrafluoroethylene (PTFE), in conjunction with the toughening agent, facilitated the construction of a dual semi-interpenetrating network. The strengthened intermolecular interactions restricted the relative slippage and mobility of the polymer chains and therefore enhanced the strength and toughness of the material. This study provides new possibilities and approaches for optimizing the comprehensive properties of bio-based polyamide materials.

## 1. Introduction

Aliphatic polyamide, as an important semi-crystalline engineering thermoplastic, has garnered widespread application in machinery, automotive, electrical appliances, and other diverse fields because of its exceptional mechanical strength, superior wear resistance, and excellent resistance to chemical solvents. Nevertheless, traditional commercial polyamides such as PA6 and PA66 are mainly derived from petroleum-based raw materials, which is contrary to the concept of environmental friendliness and sustainable development advocated today. Consequently, their widespread application is constrained. In addition, there are still some significant problems with the performance of these materials, such as strong water absorption, poor dimensional stability, and weak electrical properties, which further restrict their application in some fields [1,2,3].

In recent years, the emergence of bio-based long carbon chain polyamides (LCPA) has made up for the above shortcomings of traditional polyamides. The raw materials for bio-based LCPA are mainly derived from renewable biomass, particularly non-edible plants like castor. These raw material sources are stable and inexpensive, with the advantages of being renewable, biodegradable, and effectively reducing the impact on the environment, e.g., reduced carbon emissions. The unique structure of LCPA, containing a long methylene chain in its main chain, leads to a reduction in hydrogen bond density, thereby showing excellent toughness, low water absorption, and remarkable dimensional stability. Therefore, bio-based LCPA has been widely used in many fields [4,5,6,7,8]. However, it is worth noting that bio-based LCPA, as a semi-crystalline polymer material, has notch sensitivity, which has a significant impact on its impact resistance. The defect or tip notch becomes the area of stress concentration, which easily leads to brittle fracture. Especially in the low-temperature environment, the low-impact resistance of LCPA is more prominent, and the crack growth is difficult to control [9,10,11]. To solve this problem, over the past few decades, researchers have worked to reduce the notch sensitivity and enhance the impact resilience of LCPA. A prevalent strategy involves the incorporation of a secondary component within the matrix, which may encompass organic particulates, elastomers, or inorganic fillers [12,13,14,15,16,17,18,19,20]. The introduction of these additives is designed to enhance the toughness of materials to cater to the diverse requirements of application scenarios.

The process of enhancing the toughness of polyamide composites through the incorporation of rubber is a widely adopted and proven approach in materials science. Toughness, broadly defined, refers to a material’s ability to absorb energy and resist fracture under stress or impact. By blending rubber into the polyamide matrix, the overall ductility and resilience of the composite can be significantly improved, making it more suitable for applications where durability and impact resistance are paramount. However, simply mixing rubber particles into the polyamide base material is insufficient to achieve the desired level of toughening. The key lies in optimizing the distribution and interaction of the rubber particles within the composite structure. This is where percolation theory becomes a crucial analytical tool, providing a mathematical and conceptual framework to understand and predict the toughening mechanisms at play [21]. Percolation theory is a branch of statistical physics and network science that deals with the sudden onset of connectivity within a system as a certain parameter, such as the concentration of a component, reaches a critical threshold [22,23]. In the context of rubber-toughened polyamide composites, this theory explains how the material’s mechanical properties undergo dramatic changes when the rubber particles reach a specific density or distribution pattern within the matrix, known as the percolation threshold. To achieve optimal toughening, it is imperative that the rubber toughening agent forms a percolating network within the composite. This network should be continuous and well-dispersed, allowing for efficient stress transfer between the rubber particles and the polyamide matrix. When the rubber content reaches the percolation threshold, the network becomes sufficiently interconnected to effectively dissipate energy and prevent crack propagation, thereby enhancing the toughness of the material.

In addition, the interaction between rubber toughener and matrix material has a decisive influence on the toughening effect. If the compatibility of the two is poor, the rubber toughener may form an isolated phase region, which is not only unable to effectively transfer stress but may become a weak link in the material, leading to a decline in overall performance. Therefore, when selecting a rubber toughener, its performance characteristics, compatibility with the matrix material, and interface combination should be considered comprehensively. To ensure that the rubber toughener can be evenly dispersed in the matrix, form a good interface combination with the matrix, and then give full play to its toughening effect, in-depth research is needed in the selection of the toughening agent, the determination of the addition amount, and the optimization of the interaction between it and the matrix. In this way, it can be ensured that the method of toughening and modification by adding rubber can truly achieve the expected toughening effect.

In addition to relying on the percolation network of the toughener to achieve the toughening effect, other types of network structures can also be constructed to form an interpenetrating network structure with the percolation network of the toughener to synergistically enhance the toughness of the material. The construction of an interpenetrating network structure refers to the interweaving and interpenetrating of two or more polymers at a molecular scale to create a complex three-dimensional network. Within this network, each polymer maintains its inherent characteristics while simultaneously intertwining with other polymers. This entangled relationship facilitates a synergistic action among the polymers, enabling them to jointly resist external forces and collectively enhance the overall properties of the material. Consequently, the construction of an interpenetrating network structure within the system offers a viable means to elevate its mechanical properties, heat endurance, and dimensional stability. Furthermore, this structure enhances the damping capacity of the material, allowing it to absorb more impact energy and further optimizing its mechanical characteristics [24,25,26].

In addition to interface compatibility, in rubber toughening systems, the improvement in toughness is often accompanied by a significant decline in strength and modulus [27]. Semi-aromatic polyamide is made by copolymeizing aliphatic dibasic acid with aromatic diamine or aliphatic dibasic amine with aromatic dibasic acid. Due to the introduction of aromatic rings in the molecular chain, the heat resistance and mechanical properties are greatly improved, and the strength and modulus loss caused by the toughener can be compensated. Maximizing LCPA’s advantages and satisfying high-toughness demands necessitates investigating LCPA composite alloy compatibility. Therefore, a detailed analysis examined resin compounding and toughening agents’ impacts on the mechanical properties, focusing on type, content, and matrix interfacial interactions.

To fully capitalize on the advantages of bio-based LCPA and cater to the demand for enhanced toughness in specific applications, it is necessary to delve into the compatibility of bio-based LCPA composite alloys. Therefore, a comprehensive investigation was undertaken to study the effects of resin compounding, toughener type and amount, as well as interfacial interaction, on the mechanical behavior of the composite system.

## 2. Materials and Methods

### 2.1. Materials

**Figure 1:**
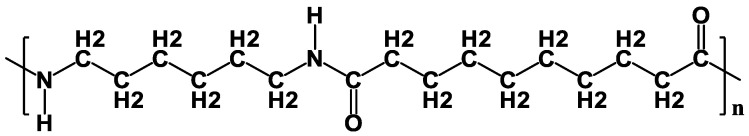
The structures of the repeating units in PA610.

**Figure 2:**
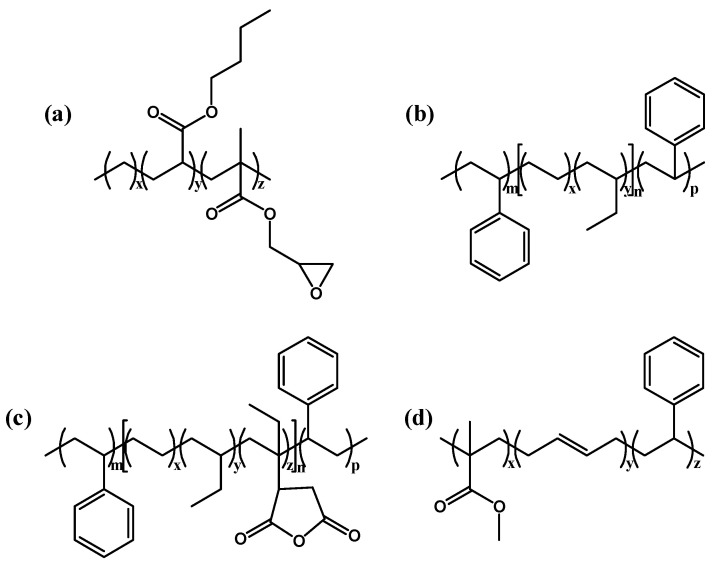
The structure formulas of the toughener (**a**) PTW, (**b**) SEBS, (**c**) MAH-g-SEBS, and (**d**) MBS.

Bio-based PA610 was prepared by polymerization of sebacic acid made from castor oil and hexanediamine and purchased from Shandong Guangyin New Material Co., Ltd. (Zibo, China). The toughener ethylene terpolymer (PTW), polystyrene-b-poly(ethylene-butylene)-b-polystyrene (SEBS), maleic anhydride-g-polystyrene-b-poly(ethylene-butylene)-b-polystyrene (MAH-g-SEBS), and poly(methyl methacrylate)-polybutadiene-polystyrene (MBS) are derived from DuPont (America), Huiju Chemical Industry Co., Ltd. (Zhengzhou, China), TSRC Corporation (Taiwan, China), and Kaneka Corporation (Tokyo, Japan), respectively. The repeating units of PA610 and the tougheners are displayed in Figure 1 and Figure 2, respectively. The primary physical parameters of PA610 are displayed in Table 1. Ultra-high-molecular-weight polytetrafluoroethylene (PTFE), with an average molecular weight of 1,000,000 g/mol, was purchased from Shandong Dongyue Polymer Materials Co., Ltd. (Zibo, China). The antioxidant, Rianlon^®^ 1098, was purchased from Rianlon Corporation (Tianjin, China). Lubricant TR044W (hyper-branched polyester) was purchased from Shanghai Brightfield Chemical Co., Ltd. (Shanghai, China).

### 2.2. Preparation of Samples

PA610 and the toughener PTW were dried at 100 °C for 8 h in a vacuum environment. The PA610/PTW/additives composite system with weight ratios of 99.3/0/0.7, 94.3/5/0.7, 89.3/10/0.7, 84.3/15/0.7, 79.3/20/0.7, 74.3/25/0.7, and 69.3/30/0.7 was fabricated utilizing a twin-screw extruder operating at 230 °C according to T_m_ with a screw speed of 300 r/min. To facilitate processing, 0.5 wt% Irganox^®^ 1098 and 0.2 wt% lubricant were also added. The prepared composites were vacuum dried at 100 °C and injection molded into standard ISO mechanical bars for tensile, flexural, and impact testing. Disk specimens (25 mm diameter and 1.0 mm thickness) prepared through the melting and pressing process under 230 °C and 30 MPa for 3 min and then cooled to room temperature by cold compression cooling were used for rheology tests.

Using the same processing technology, the dried PA610 and PTFE were mixed and granulated by a twin-screw extruder, in which the PTFE content was 0 wt%, 0.3 wt%, 0.5 wt%, to fabricate PA610/PTFE composites. Additionally, the composites of PA610 with different tougheners were prepared according to the formula detailed in Table 2. Standard ISO mechanical bars and disk specimens were also prepared for mechanical and rheology tests.

### 2.3. Characterization Methods

Tensile and flexural properties were evaluated adhering to the ISO 527-2-2012 standard [29], with testing conducted at speeds of 10 and 2 mm/min, respectively. The Izod impact strength measurements were tested at 24 °C by using a 2.75 J pendulum according to the ASTM D256-23e1 standard [30]. To maintain data reliability and accuracy, each mechanical property test was reiterated at least 5 times.

The rheological properties of composites were characterized using a stress-controlled rheometer (DHR-2, TA Instruments, USA) with a parallel plate geometry (25 mm diameter and 0.8 mm sample gap). To prevent sample degradation, the sample chamber was continuously purged with nitrogen flow. Initially, a strain sweep test was conducted at 1 Hz to determine the liner viscoelastic regime. According to the experimental results, the oscillatory frequency sweep experiment was performed at a strain amplitude of 1% ranging from 628 to 0.01 rad/s. Additionally, the stress relaxation test was at a strain amplitude of 1% and 280 °C.

## 3. Results and Discussion

### 3.1. Rheological Behavior and Interfacial Interaction of Composite Systems

Rheological analysis is a powerful technique to investigate the rheological properties of materials, particularly in examining the influence of the kind and amount of toughener on composite characteristics. Key rheological parameters, including viscosity, elasticity, and loss factor, are affected by the kind and amount of toughener. These changes can be measured by rheological analysis to assess the impact of toughener composition and content on properties.

**Figure 3:**
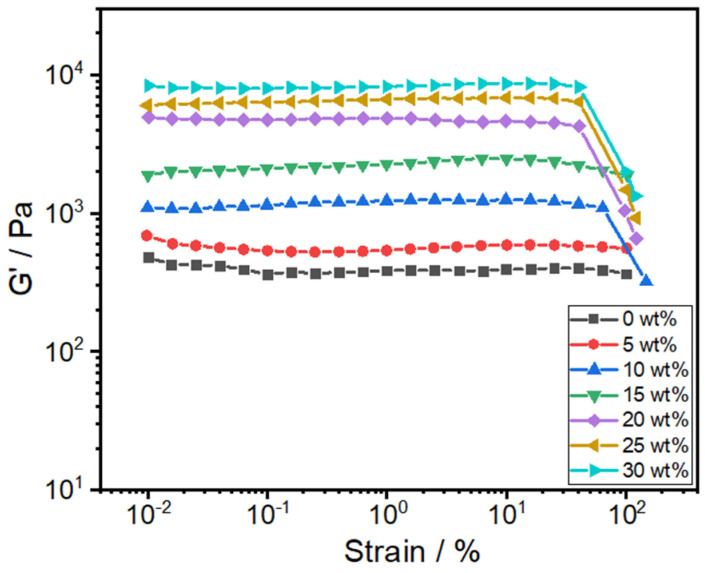
Storage modulus (G′) of PA/PTW composites as a function of strain amplitude at 1 Hz and 280 °C.

In the linear viscoelastic regime, variations in storage modulus (G′), loss modulus (G″), and complex viscosity (η*) as functions of angular frequency could serve as indicators to verify the formation of a percolation network structure [31,32]. To assess the percolation network concentration of toughener PTW in matrix PA610, the rheological properties of composites were characterized at 280 °C. In order to ensure that the sample did not degrade during the tests, the isothermal TGA at 280 °C for 3 h and the oscillatory shear temperature sweep test were first performed to prove the thermal stability of the sample at a high temperature of 280 °C (Figure S2). The linear viscoelastic regime was determined through amplitude scanning at 1 Hz for all samples (Figure 3). The frequency sweep test, ranging from 628 to 0.01 rad/s, was conducted at a strain amplitude of 1%.

**Figure 4:**
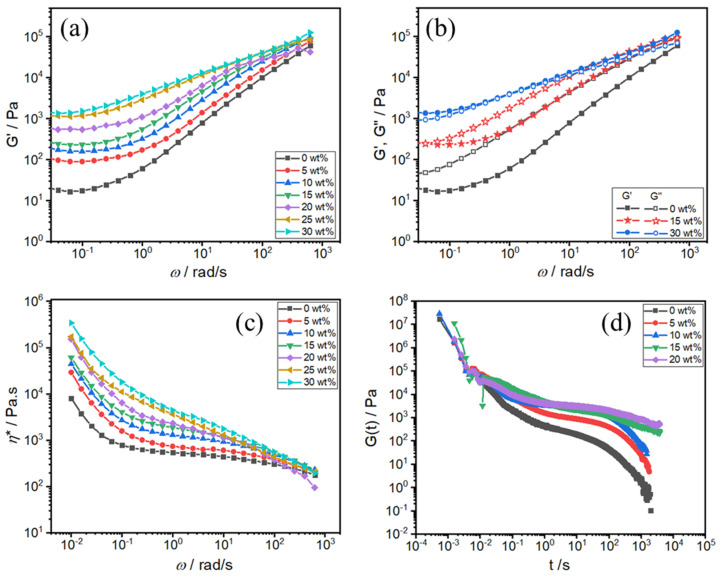
(**a**) Storage modulus (G′), (**b**) comparison of storage modulus G′ (solid symbols) and loss modulus G″ (hollow symbols), (**c**) complex viscosity (η*), and (**d**) modulus of the melt with respect to time during the stress relaxation of PA/PTW composites.

Figure 4 presents a comparative analysis of the effects of toughener PTW on G′, G″, and η* relative to neat PA610. As the PTW content increases, there are notable enhancements in the values of G′, G″, and η* across the entire frequency range. Notably, a plateau emerges in the low-frequency region (Figure 4a). A plateau appears in the low-frequency region of pure PA systems, which is attributed to the inherent characteristics of PA. To obtain high-molecular-weight PA resin, its preparation process involves two steps: melt polymerization and solid-phase viscosification. During this solid-phase viscosification process, a portion of crosslinking or gel formation takes place within the system, causing it to deviate from the scaling law in the linear viscoelastic regime (G′-ω^2^, G″-ω^1^). This deviation has been thoroughly described in our previous research work [33]. This plateau appears earlier as the PTW content increases from high frequency to low frequency, reflecting the polymer chain’s long-range motion constraints. The presence of the plateau signifies incomplete relaxation of the polymer chains, indicating network structure formation [33,34,35]. Furthermore, the transition from liquid-like (G′ < G″) to solid-like (G′ > G″) behavior in the composites is discernible in Figure 4b. In the low-frequency range, as PTW content increases, the log–log plots of G′ versus ω and G″ versus ω gradually converge, with G′ eventually exceeding G″, underscoring the enhanced solid-like behavior and confirming the establishment of a network structure.

**Figure 5:**
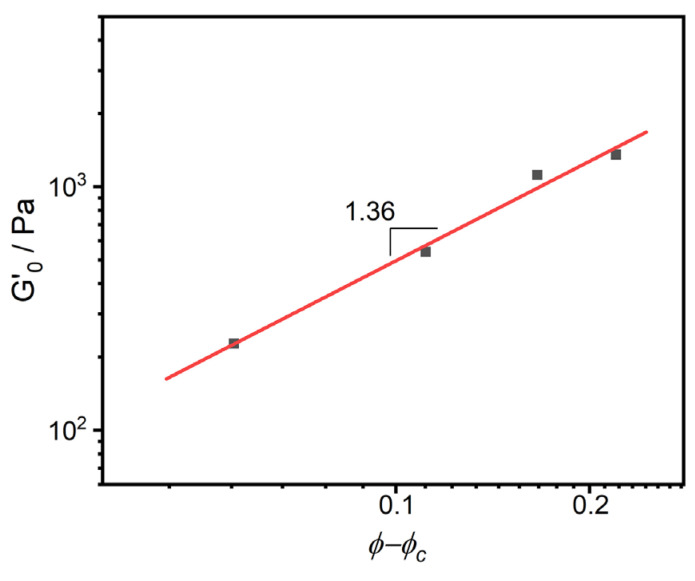
Power law relation of modulus G_0_′ to volume fraction *ϕ-ϕ_c_*.

The network structure is divided into a chemical network and a physical network. The physical network arises from entanglement, which occurs when the percolation concentration is reached with the increase in toughener content, and can be completely relaxed over a sufficiently long time. The chemical networks emerge from cross-linking or chemical interactions; therefore, they are difficult to relax completely [11,21]. From Figure 4d, it can be observed that when the toughener content exceeds the percolation threshold of 15 wt%, the stress does not fully relax after a long time, which is due to the presence of good chemical interactions between the toughener and the polyamide matrix; that is, the epoxy groups in the toughener PTW produce chemical reactions with the terminal carboxyl and amino groups of PA610, leading to the formation of a chemical network structure. Therefore, in this system, when the concentration of toughener exceeds the percolation threshold, there are physical entanglement networks and chemical networks. This network structure architecture effectively dissipates fracture impact energy, arrests the progression of micro-cracks, and consequently enhances the material’s impact property. Combining the trend of mechanical properties with the variation of toughener content (Figure S3) and the appearance of the low-frequency plateau in rheological properties, the percolation network concentration *ω*_c_ is determined to be 15 wt%. According to the percolation theory [36], when the particle volume fraction *ϕ* exceeds the percolation concentration *ϕ*_c_, the strength of the percolation network satisfies the relationship:

(1) $$G_{0}^{\prime }=K{\left(\emptyset -\emptyset_{c}\right)}^{v}$$

(2) $$\phi_{PTW}=\frac{\omega_{PTW}\rho_{PA610}}{\omega_{PTW}\rho_{PA610}+\left(1-\omega_{PTW}\right)\rho_{PTW}}$$

where G_0_′, defined as the modulus that is invariant to *ω*, is derived from the low-frequency plateau region of the storage modulus in the rheological characterization, representing an intrinsic material property. The parameters *k* and *v* are utilized as fitting coefficients. *ϕ*, *ω,* and *ρ* represent the volume fraction, weight fraction, and density, respectively. *ρ*_PTW_ = 0.94 g/m^3^, *ρ*_PA610_ = 1.06 g/m^3^. When the polymer matrix exhibits strong affinity with filler particles, the value of *v* remains low; otherwise, the value of *v* is higher; that is, the value of *v* is contingent on the interaction occurring between the polymer matrix and the dispersed particles [37,38]. This is also consistent with the previous research conclusion [39,40], which posits that in the presence of a chemical interplay between the matrix and the filler, the value of *v* approximates 1.88. Alternatively, when only a physical network exists between them, the value of *v* spikes to 5.3. Notably, the PA/PTW system exhibits a value of 1.36 (Figure 5), suggesting excellent compatibility between the polyamide matrix and the toughener filler PTW.

**Figure 6:**
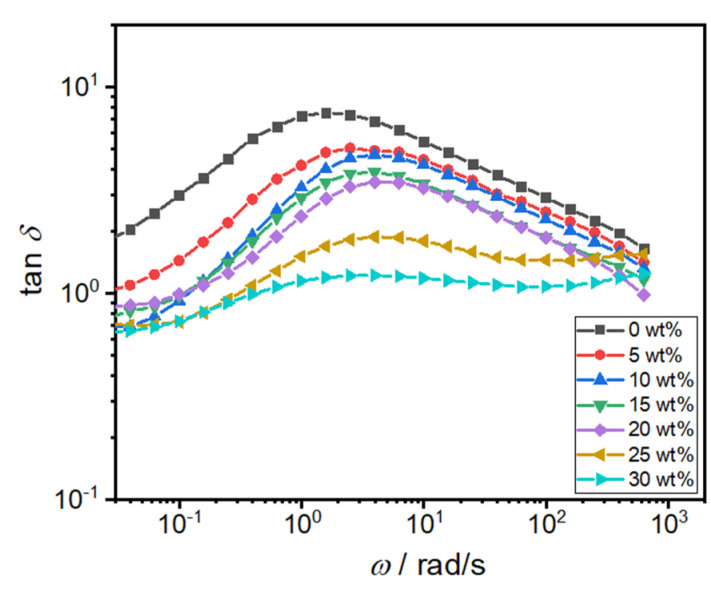
The plots of tan *δ* vs. *ω*.

The strong interfacial interaction between PTW and PA facilitates the efficient transfer of internal stress from the polymer matrix to the toughener particles. The tan *δ*, the tangent of the ratio between the loss modulus G″ and the storage modulus G′, serves as an indicator of the relative proportion of energy loss to energy storage in a material under alternating stress conditions. Consequently, tan *δ* is frequently employed as a metric to characterize the damping properties of a material, encapsulating the energy dissipation arising from friction within molecular chain motions [11]. A lower tan *δ* value suggests that when the material is subjected to external forces, the incorporation of tougheners effectively constrains molecular chain mobility, impeding energy dissipation and enhancing the system’s elastic response. The tendency for tan *δ* to exhibit a flatter profile implies that the material can demonstrate superior damping performance across a broader frequency spectrum. Illustrated in Figure 6, the diminishing trend of tan *δ* with an increase in toughener content suggests that the PA/PTW system exhibits superior elastic response, attributed to the obstruction of energy dissipation by the network structure.

### 3.2. Selection of Toughener in Composite System

**Figure 7:**
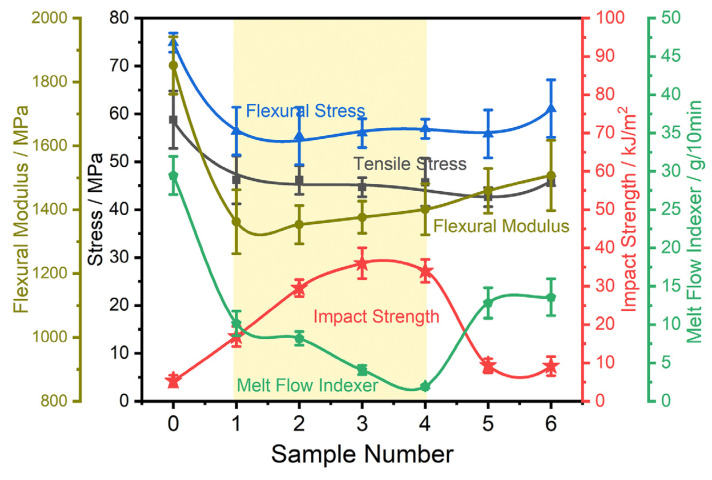
Mechanical properties of different polyamide toughening systems (the numerical labels indicating the following compositions: 0 represents pure PA, 1 represents PA and 15 wt% PTW, 2 represents PA and 10 wt% PTW and 5 wt% MAH-g-SEBS, 3 represents PA and 5 wt% PTW and 10 wt% MAH-g-SEBS, 4 represents PA and 15 wt% MAH-g-SEBS, 5 represents PA and 15 wt% SEBS, 6 represents PA and 15 wt% MBS. The numerical prefixes represent the mass fractions of the respective tougheners).

The four types of tougheners shown in Figure 2 were extruded according to Table 1. An investigation was conducted on the mechanical properties and rheological characteristics of these toughened composite systems. Figure 7 illustrates the mechanical properties of different polyamide toughening systems. By incorporating tougheners, the toughness (measured by notched impact strength) of composite systems can be significantly enhanced, albeit with a compromise in strength and modulus. Consequently, the selection and adjustment of toughener must be tailored to specific application requirements. Among the various toughening systems, PTW, MAH-g-SEBS, and their combination exhibit superior toughness. This enhancement stems from the presence of polar groups in the toughener, which enhance compatibility, facilitate efficient stress transduction from the PA to the toughener, and ultimately improve toughness. Notably, MAH-g-SEBS demonstrates a superior toughening effect on polyamide compared to the ungrafted SEBS. Although the core–shell particle MBS incurs litter strength loss to the composite system, its toughening effect is negligible. Considering their mechanical and flowing properties, PTW, MAH-g-SEBS, and their combination of toughening systems emerge as the most effective options.

During actual production, in addition to mechanical properties, the viscosity of the system should also be taken into account. The increase inviscosity of the system will significantly affect its rheological characteristics during processing, causing fluidity to decline and processing complexity to increase, which will limit the popularization and application of the system in practical applications. This constraint not only decelerates production efficiency but may also adversely affect product quality and performance. Therefore, it is urgent to investigate optimization techniques aimed at enhancing processing capabilities and expanding the application scope.

**Figure 8:**
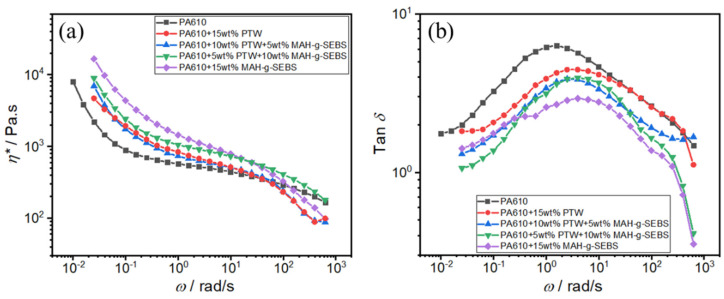
(**a**) the complex viscosity η*, (**b**) the loss factor tan *δ* of PA composite systems with different tougheners as a function of angular frequency *ω*.

Based on the above research results, PTW, MAH-g-SEBS, and PA matrix exhibit favorable interactions. The toughener MAH-g-SEBS particularly has a noteworthy toughening effect; however, the fluidity of the system is poor. The toughener PTW, on the other hand, exhibits a secondary toughening effect while maintaining superior system fluidity. To assess the fluidity of PTW, MAH-g-SEBS, and their mixed systems, the complex viscosity η* within rheological characterization (Figure 8a) was employed. The results indicate that combinations of MAH-g-SEBS and PTW could enhance system fluidity, that is, 15 wt% PTW > 10 wt% PTW + 5 wt% MAH-g-SEBS > 5 wt% PTW + 10 wt% MAH-g-SEBS > 15 wt% MAH-g-SEBS. Figure 8b illustrates the relationship between the loss factor tan *δ* and angular frequency *ω* of PA composite systems with different tougheners. Tan *δ* is often used to represent the damping properties of a material and reflects the energy consumption due to molecular chain motion friction. Tan δ, which represents the viscoelastic behavior of the material, becomes smaller, indicating that the addition of tougheners significantly restricts molecular chain movement, hinders the dissipation of energy, and enhances the system’s elastic behavior. Notably, as MAH-g-SEBS content increases in the toughened system, the tan *δ* value decreases, which is consistent with the previous mechanical data. Consequently, the employment of tougheners effectively enhances fluidity while maintaining system toughness.

### 3.3. Construction of Interpenetrating Network Structure

**Figure 9:**
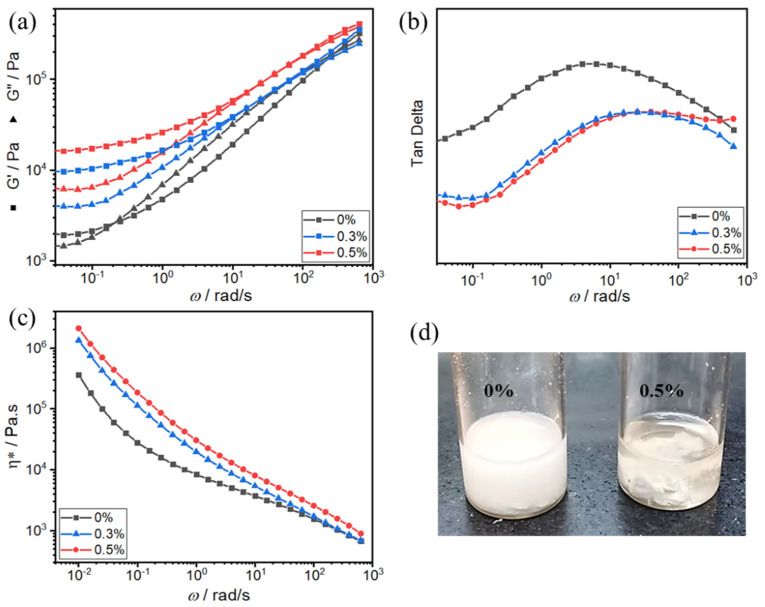
(**a**) Comparison of the storage modulus (G′) and the loss modulus (G″), (**b**) the loss factor (tan *δ*) and (**c**) the complex viscosity η* as a function of angular frequency (*ω*), and (**d**) the dissolution experiment diagram in TFA of pure PA610 and PA610/PTFE composite systems.

Network construction in systems could elevate mechanical properties. Beyond the toughener’s percolation network, diverse network architectures can be devised to intertwine with it, forming an interpenetrating network that collaboratively enhances the material’s toughness. In this study, a semi-interpenetrating network structure was constructed by adding the second component of ultra-molecular-weight polytetrafluoroethylene (PTFE). Xu et al. [41] observed and confirmed the presence of PTFE nanofiber networks by expanding the PA6 matrix from the fibers during gas nucleus growth using foam generation techniques. There are two networks in the system: one is the percolation network formed by the toughener in the matrix, and there is a chemical interaction between the toughener and PA; and the other is the fibrillation of high-molecular-weight PTFE during the extrusion process, forming a linear chain interpenetration. In order to verify the formation of the network structure, the dissolution experiment was carried out. PA610 can be dissolved in the polar solvent trifluoroacetic acid (TFA), while ultra-high-molecular-weight PTFE is difficult to dissolve. When PA610 and a blend of PA610 with 0.5 wt% PTFE (PA610 + 0.5 wt% PTFE) were exposed to TFA, distinct behaviors were observed (Figure 9d). After standing for 5 h, PA610 was fully dissolved, whereas the blend exhibited swelling without complete dissolution. This observation suggests that PTFE forms a network structure in the composite material and provides an effective support for the matrix.

Within the linear viscoelastic region, variations in the storage modulus (G′), loss modulus (G″), and complex viscosity (η*) with respect to angular frequency *ω* afford insights into the existence of a network structure [37]. To elucidate the formation of an ultra-high-molecular-weight PTFE fibrillation network within the PA matrix, the rheological characteristics of the composite were systematically analyzed at 280 °C. Initially, an amplitude sweep test was conducted at a fixed frequency of 1 Hz across all samples to delineate the linear viscoelastic region. Subsequently, frequency sweep tests were executed spanning a frequency range from 628 to 0.01 rad/s, while maintaining a constant strain of 5%, to further investigate the rheological behavior.

Compared with pure PA610 systems, the effects of ultra-high-molecular-weight PTFE on the storage modulus (G′), loss modulus (G″), and complex viscosity (η*) of systems are shown in Figure 9a,c. As the PTFE content increases, notable enhancements in G′, G″, and η* are observed across the entire frequency range. Figure 9a depicts a discernible plateau formation in the low-frequency region upon achieving a PTFE concentration threshold of 0.3 wt%. With the increase in PTFE content, the time of the emergence of the plateau from high to low frequency is advanced. Rheological analysis interprets the low-frequency region as a reflection of long-range polymer chain motion. The emergence of the plateau phenomenon in the rheological response indicates incomplete relaxation dynamics within the polymer chains, suggesting the establishment of a network structure. Additionally, when G′ is inferior to G″, the composite system exhibits liquid-like characteristics; conversely, the prevalence of G′ over G″ signifies solid-like behavior. Figure 9a reveals that, within the low-frequency region, an increase in PTFE content leads to a gradual convergence of the log–log plots of G′ vs. *ω* and G″ vs. *ω*, ultimately resulting in G′ surpassing G″. This transition indicates the enhancement of solid-like attributes and provides evidence for the formation of the network structure. The network structure formed by PTFE in the PA610 matrix enhances internal stress transfer and prevents the further propagation of micro-cracks. Tan *δ* reflects the damping characteristics of the material. Figure 9b illustrates that tan *δ* decreases with increasing PTFE content, indicating that the composite system exhibits better elastic behavior due to the obstruction of energy dissipation by the network structure.

**Figure 10:**
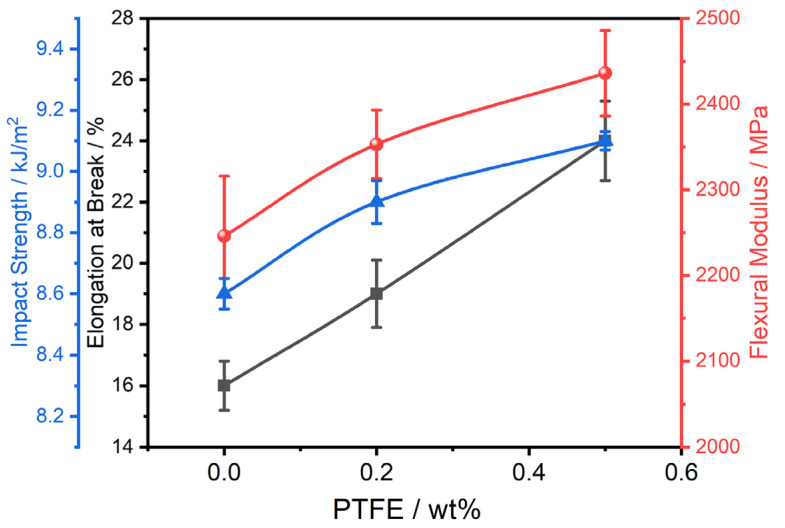
Mechanical properties of systems with different contents of ultra-high-molecular-weight PTFE.

The rheological behaviors are consistent with the mechanical properties. As Figure 10 illustrates, a comparative analysis of the tensile, flexural, and impact properties of pure PA610 and PA610/PTFE composite systems reveals that the inclusion of PTFE positively impacts both the flexural modulus and the notch impact strength. This observation suggests that the formation of an interpenetrating network structure effectively disperses and transfers loads during stress application, thereby enhancing the modulus and toughness of the composite material. In summary, the addition of ultra-high-molecular-weight PTFE to construct an interpenetrating network structure significantly improves the mechanical properties of the composite.

## 4. Conclusions

In this study, the dual interpenetrating toughening network of the percolation network of the toughener and the ultra-high-molecular-weight PTFE has been collaboratively constructed in the PA610 matrix, and its toughening effects have been investigated comprehensively. In the rheology study, the existence of a low-frequency platform with a percolation concentration of 15 wt% within the network and the interfacial interaction between PA 610 and toughener particles quantified as *v* = 1.36 using percolation theory indicate good compatibility and affinity between them. Due to the strong interfacial interaction between the matrix and the toughener with polar groups, the internal stress transfers from the matrix domains to the toughening domains, thereby enhancing the toughness. Furthermore, the rheological and melt flow index analysis revealed that the mechanical properties and fluidity of this blend system can be optimized effectively by different tougheners. The network of ultra-high-molecular-weight PTFE in conjunction with the toughener strengthens the interaction between the molecular chains, which limits the relative slip and chain mobility and consequently leads to a significant improvement in both strength and toughness of the blend system.

## Figures and Tables

**Table 1:** Primary physical parameters of PA610.

	^a^ MFI (g/10 min)	^b^ T_c_ (°C)	^b^ T_m_ (°C)	^c^ T_peak_ (°C)
PA610	23.7	179.9	222.9	481.9

^a^ Determined by melt flow indexer (MFI) at 235 °C under a load of 2.16 kg according to ASTM D1238 [28]. ^b^ Determined by differential scanning calorimetry (DSC TA Q200, TA Instruments, New Castle, DE, USA, Figure S1a). ^c^ Determined by thermal gravimetric analysis (TGA, PE Pyris 1, Perkin Elmer, Waltham, MA, USA, Figure S1b).

**Table 2:** The formula of composites with PA610 and different tougheners.

	1#	2#	3#	4#	5#	6#	7#
PA610	99.5	84.5	84.5	84.5	84.5	84.5	84.5
PTW		15	10	5			
MAH-g-SEBS			5	10	15		
SEBS						15	
MBS							15
Irganox^®^ 1098	0.5	0.5	0.5	0.5	0.5	0.5	0.5
TR044W	0.2	0.2	0.2	0.2	0.2	0.2	0.2

## Data Availability

The original contributions presented in the study are included in the article/Supplementary Material, further inquiries can be directed to the corresponding author.

## Supplementary Materials

The following supporting information can be downloaded at: https://www.mdpi.com/article/10.3390/polym16162248/s1, Figure S1. (a) Secondary heating curves and cooling curves, (b) The thermal gravity analysis (TGA) curves (inset: the derivative thermal analysis curves) of PA610; Figure S2. (a) The isothermal thermal gravimetric analysis and (b) the oscillatory thermal temperature sweep at 280 °C; Figure S3. The impact strength and melt flow indexer vs. PTW content.
