# Supplementary material for: Investigation of Dual Network Construction for Toughening in Bio-Based Polyamide Composites
Source: Polymers (Basel). 2024 Aug 8;16(16):2248. doi: 10.3390/polym16162248 (PMC11359592; doi:10.3390/polym16162248)
Supplement: Supplementary file 1 [file polymers-16-02248-s001.zip › polymers-3089314-supplementary.pdf]

Investigation of Dual Network Construction for Toughening in Bio-based Polyamide Composites

Chenxu Zhou [1,2], Chao Ding [1,3], Huaguang Yang [1], and Xianbo Huang [1*]

[1] National Engineering Laboratory for Plastic Modification and Processing, Kingfa Scientific and Technological Co. Ltd., Guangzhou 510275, China

[2] College of Polymer Science and Engineering, State Key Laboratory of Polymer Materials Engineering of China, Sichuan University, Chengdu 610065, China

[3] Institute of Emergent Elastomers, School of Materials Science and Engineering, South China University of Technology, Guangzhou 510641, China

[*] Correspondence: author: E-mail: huangxianbo@kingfa.com.cn (X.B.H.)

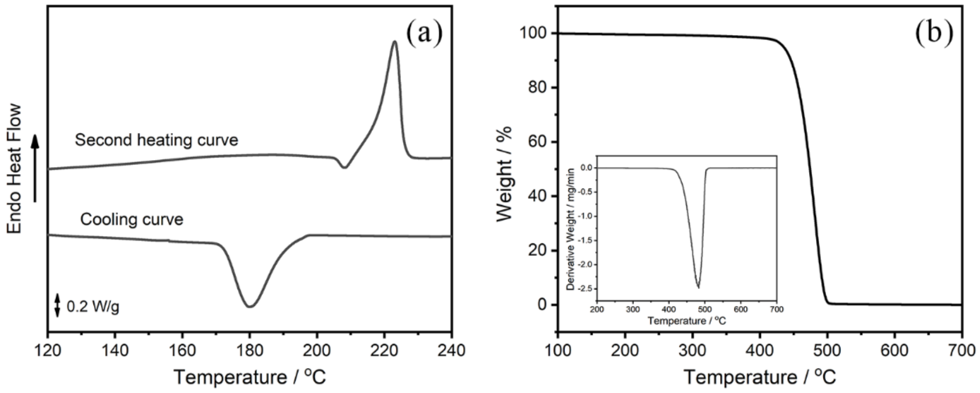

Figure S1. (a) Secondary heating curves and cooling curves, (b) The thermal gravity analysis (TGA) curves (inset: the derivative thermal analysis curves) of PA610.

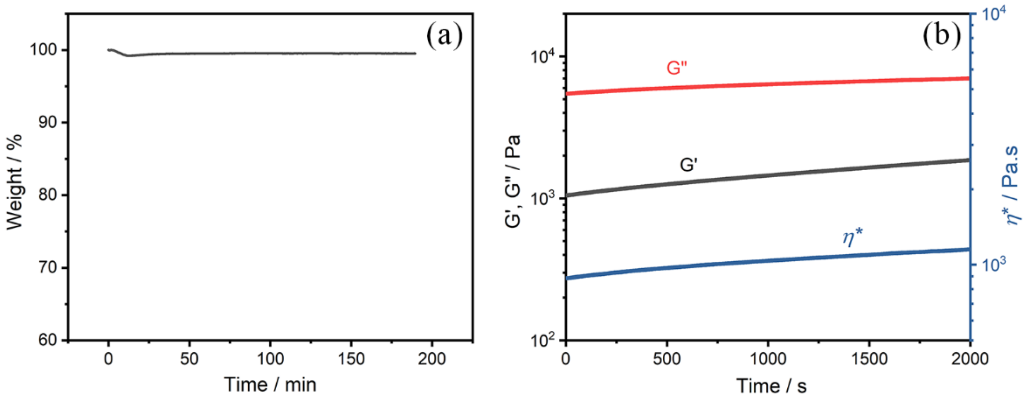

Figure S2. (a) The isothermal thermal gravimetric analysis and (b) the oscillatory thermal temperature sweep at 280 °C.

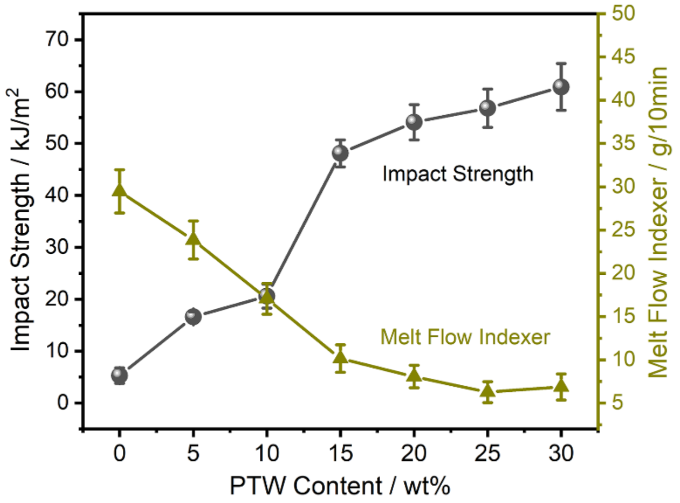

Figure S3. The impact strength and melt flow indexer *vs*. PTW content.
